# Extrahepatic Autoimmune Diseases in Patients with Autoimmune Liver Diseases: A Phenomenon Neglected by Gastroenterologists

**DOI:** 10.1155/2017/2376231

**Published:** 2017-01-16

**Authors:** Liping Guo, Lu Zhou, Na Zhang, Baoru Deng, Bangmao Wang

**Affiliations:** ^1^Department of Gastroenterology and Hepatology, Tianjin Medical University General Hospital, Tianjin 300052, China; ^2^Department of Rheumatology, Tianjin Medical University General Hospital, Tianjin 300052, China

## Abstract

Autoimmune liver diseases (AILDs) often coexist with other extrahepatic autoimmune diseases (EHAIDs). The spectrum of EHAIDs in patients with AILDs is similar, whereas the incidence is different. Notably, autoimmune thyroid disease and Sjogren's syndrome are the most common EHAIDs. Associated extrahepatic diseases may predate the appearance of AILDs or coincide with their onset. More frequently, they may appear during the course and even occur years after the diagnosis of AILDs. Importantly, associated EHAIDs may influence the natural course and prognosis of AILDs. To date, a definite pathophysiological pathway which contributes to the coexistence of AILDs and EHAIDs is still lacking. The current view of autoimmunity clustering involves a common susceptibility genetic background which applies to related pathologies. Herein, we review the current published researches regarding EHAIDs in patients with AILDs, particularly in relation to their clinical impact and pathophysiology. In managing patients with AILDs, gastroenterologists should be aware of the possibly associated EHAIDs to ensure a prompt diagnosis and better outcome.

## 1. Introduction

Autoimmune liver diseases (AILDs) fall into two broad categories, those with hepatic predominance such as autoimmune hepatitis (AIH) and those with predominance of cholestatic features such as primary biliary cirrhosis (PBC) and primary sclerosing cholangitis (PSC). The concurrence of clinical, biochemical, serological, and/or histological features suggesting AIH and PBC (or PSC) has been described as overlap syndrome (OS). Importantly, AILDs also coexist with other extrahepatic autoimmune diseases (EHAIDs) and conditions, thereby causing not only liver damage, but also extrahepatic injury. However, the relationship between AILDs and concomitant EHAIDs is strongly debated. Currently, two hypotheses exist: (i) it is thought that AILDs are part of multiple organ involvement in a systemic autoimmune disease, particularly in non-organ specific autoimmune diseases such as systemic lupus erythematosus (SLE), rheumatoid arthritis (RA), systemic sclerosis (SSc), and Sjogren's syndrome (SS); (ii) it is thought that AILDs and concomitant extrahepatic autoimmune conditions are different disorders, linked by a common pathogenetic pathway, for example, the coexistence of AILDs and chronic lymphocytic thyroiditis, hyperthyroidism, myasthenia gravis, or pernicious anemia. Herein, we discuss EHAIDs in patients with AILDs, particularly in relation to their clinical impact and pathophysiology.

## 2. The Spectrum of EHIADs in Patients with AILDs

The spectrum of EHAIDs in patients with AILDs is similar in current reports, whereas the incidence is different (Table 1). Notably, autoimmune thyroid disease (AITD) and SS are the most common EHAIDs in patients with AILDs. Associated extrahepatic diseases may predate the appearance of AILDs or coincide with their onset. More frequently, they may appear during the course and even occur years after the diagnosis of AILDs. Watt et al. [1] reported that 84 of 160 (53%) PBC patients had at least one additional extrahepatic autoimmune condition, and 16 of 37 (43%) patients developed thyroid diseases prior to the detection of PBC, while 19 of 37 (51%) patients detected thyroid diseases at the same time or following the diagnosis of PBC; 10 of 12 (83%) patients developed sclerosis symptoms prior to the detection of PBC. Another report also showed that the onset time of AIH and dermatomyositis (DM) was uneven [2]. While explanations for the discrepancies in incidence and onset time are lacking, different geographical and genetic backgrounds in studies may be involved.

## 3. Nonspecific Liver Involvement or AILDs in the Course of EHIADs

Autoimmunity clustering frequently increases the difficulty of diagnosis. It is clinically important for gastroenterologists to early screen patients with AILDs for concomitant EHIADs and to make an accurate diagnosis according to their respective diagnostic criteria. Connective tissue diseases (CTDs) are systemic disorders that have an autoimmune basis and involve multiple organs or tissues, such as liver, kidney, and lung. Indeed, patients with CTDs often have concomitant liver abnormalities; about 3.0–79.0% of patients with SLE and 7.0–49.0% of patients with SS showed liver dysfunction (Table 2). When confronted with such patients, gastroenterologists need to classify these liver abnormalities as a primary liver disease with associated autoimmune, clinical, and laboratory features or a generalized liver involvement manifestation of CTDs. The classical example of this differential diagnosis dilemma is AIH and CTDs associated hepatitis, both of which have autoimmune symptoms. Antinuclear antibody (ANA) and immunoglobulin (Ig) G are not unique in AIH, which can be also positive in CTDs. On the other hand, patients with CTDs often show liver abnormalities as mentioned before. Therefore, it is big confusion to distinguish them just according to symptoms, physical signs, and autoantibodies. Liver biopsy may be helpful in such patients. Histopathological manifestations of CTDs associated hepatitis may vary from subclinical liver diseases with nonspecific changes to chronic active hepatitis, chronic persistent hepatitis, fibrosis, cirrhosis, nodular regenerative hyperplasia, and so on [17, 21].

Otherwhile, external factors such as drugs can trigger susceptible patients with risk alleles of the major histocompatibility complex (MHC). Drug-induced autoimmune liver disease (DIAILD) refers to the latent autoimmunity with positive autoantibodies, including drug-induced liver injury (DILI), drug-induced-AIH (DI-AIH), and immunity mediated DILI (IM-DILI) [39]. It is important to remember that another main cause of biochemical liver abnormalities in patients with CTDs is drug-induced alterations. Almost all drugs in the armamentarium against SLE or other rheumatologic diseases may lead to liver toxicity, such as nonsteroidal anti-inflammatory drugs (NSAIDs). NSAID-associated liver injuries vary from slightly biochemical and histological abnormalities to severe liver fibrosis, cirrhosis, chronic liver failure, or even fulminant hepatic failure [22, 23]. A prospective study found that 28 of 260 (10.8%) patients with active SLE showed salicylate poisoning and biochemical liver abnormalities. In this study, 14 patients underwent liver biopsies, by which a nonspecific inflammatory reaction was confirmed. Additionally, immunosuppressive drugs such as antitumor necrosis factor- (TNF-) *α* were reported to induce liver injuries. The immune-mediated drug reaction in the liver must be monitored during using biologics [40]. Histological performances of AIH and DILI have certain similarities, including interface hepatitis, inflammatory cells infiltration in portal area, and centrilobular 3 zone necrosis [17, 41]. Suzuki et al. [42] compared 35 cases of DILI with 28 cases of AIH according to Ishak score, portal inflammatory cell types, penetration phenomenon, rosette, and cholestasis. The results showed that interface hepatitis, focal necrosis, and portal inflammation existed both in AIH and DILI; however, neutrophils infiltration in portal area and cholestasis were more common in DILI. In addition, Suzuki et al. [42] suggested that compared with AIH DILI had no obvious liver fibrosis. Other studies indicated that eosinophils infiltration was more common in DILI, but some findings showed that eosinophils infiltration was not conducive to distinguish DILI from AIH [31]. Therefore, in addition to medication history and clinical manifestations, liver biopsy is crucial to assist in distinguishing AILDs in the course of EHAIDs from CTDs associated or drug-induced liver injuries.

## 4. The Impact of Concomitant EHAIDs on the Natural Course and Prognosis of AILDs 

Whether the concomitant EHAIDs affect the natural course and prognosis of AILDs is unclear as only a few related researches have been carried out. In a latest study from UK, Wong et al. [6] systematically assessed features and clinical impact of EHAID on AIH. Autoimmune skin diseases were more prevalent in AIH-2 than AIH-1 (21.9% versus 7.0%, *P* = 0.009), which suggested that presence of EHAIDs might influence clinical phenotype of AIH at presentation. Personal history of EHAIDs was more commonly found in AIH patients with than without first-degree family history of EHAIDs [48/86 (55.8%) versus 169/446 (37.9%), *P* = 0.002]. AIH patients with EHAIDs were more often women (85.2% versus 76.1%, *P* = 0.008), had higher posttreatment IAIHG score (22 versus 20, *P* < 0.001), had less reactivity to smooth muscle antibodies (49.8% versus 65.0%, *P* < 0.001), were more likely to have mild fibrosis at diagnosis (20.9% versus 6.5%, *P* < 0.001), and less often had ascites (6.3% versus 13.6%, *P* = 0.008) and coagulopathy (1.18 versus 1.27, *P* = 0.013) at presentation. However, presence of EHAIDs did not significantly affect disease progression, prognosis, and survival in AIH.

Muratori et al. [43] investigated 608 Italian patients with AILDs (327 with AIH and 281 with PBC) for concomitant EHAIDs and assessed the incidence and clinical impact of associated EHAIDs on AILDs. AIH patients with EHAIDs showed significant female predominance (male/female: 63/163 versus 9/91). In addition, an EHAID was more often detected in patients with an onset of AIH devoid of any particular liver-related symptoms, and the concomitant EHAIDs did not modify the features of PBC patients.

Wang et al. [19] screened 322 Chinese PBC patients for the presence of CTDs and identified the differences in clinical features and laboratory findings between PBC patients with or without CTDs. Compared to patients with PBC alone, PBC-SLE patients had lower *γ*-glutamyl transpeptidase (*γ*-GGT) and immunoglobulin M (IgM) levels, suggesting that presence of SLE in PBC patients appeared to be associated with significantly less extensive liver damage and SLE might protect against progression of PBC by delaying cirrhosis and the need for liver transplantation; PBC-RA patients had higher serum immunoglobulin G (IgG) and alkaline phosphatase (ALP) levels, suggesting that presence of RA might be a harmful factor in the prognosis of PBC. The presence of SS, SSc, and polymyositis (PM) did not seem to have any impact on the clinical course and prognosis of PBC; however, their unique features emerged. PBC-SS patients were more likely to have fever and elevated erythrocyte sedimentation rate (ESR), a higher incidence of rheumatoid factor (RF) seropositivity, and interstitial lung disease (ILD), suggesting that patients with concomitant autoimmune disorders might have an aggravated inflammatory response; PBC-SSc patients had a higher incidence of ILD; PBC-PM patients had a higher white blood cell (WBC) count and incidence of myocardial involvement. In another study, adjusting for sex, age, log bilirubin, and ALP, the risk of transplantation or death from diagnosis was significantly lower in PBC-SSc (hazard ratio 0.116, *P* = 0.01) [44], suggesting that PBC patients with SSc had a better prognosis.

As mentioned before, the first two researches investigated the features and clinical impact of concomitant EHAIDs on AIH patients. Similar result was female predominance in AIH patients with concomitant EHAIDs. Besides analyses of some clinical features at AIH presentation and prognosis, Wong et al. [6] paid more attention to the personal and family EHAIDs history and impact of EHAIDs on AIH clinical phenotype. The other three researches all analyzed the clinical features and prognosis of PBC patients with or without concomitant EHAIDs. Wang et al. [19] found that SLE or RA might be a beneficial or harmful factor in the prognosis of PBC, and SS, SSc, and PM seemed to have no impact on the clinical course and prognosis of PBC. However, the last paper suggested that concomitant SSc significantly lowered the risk of transplantation or death in PBC patients. Taken together, different EHAIDs may have different influences on the natural course and prognosis of AILDs. Certain EHAIDs may aggravate the systemic inflammatory response and liver damage, and others may alleviate the liver inflammatory response, consequently achieving a better prognosis. Meanwhile, it is worth paying more attention to the higher incidence of ILD in autoimmune clustering. Of course, differences between the researches may be due to entirely different population backgrounds. Furthermore, some multicenter researches with a large sample size and different population background are needed to explore and verify these findings.

## 5. Common Pathophysiological Pathways in AILDs and EHAIDs 

Autoimmune diseases appear to result from a complex series of interactions between susceptibility genes, environment, and immune system. However, the researches on pathophysiological pathways of concomitant autoimmune diseases are still relatively limited. Recently, with the development of molecular genetics, human genome-wide association studies (GWAs) and risk-associated single nucleotide polymorphism (SNP) have revealed that these patterns of coexistence/overlap depend predominantly on genetic determinants [24–26, 32, 33, 35–38]. Most of these so-called susceptible gene loci are widely distributed in many autoimmune diseases and thus contribute strongly to their coexistence. The recently completed GWAs reported a significant connection between PBC and STAT4, which is also the prominent risk gene in AITD, type 1 diabetes, SLE, RA, SS, and inflammatory bowel disease (IBD) [24, 32, 36, 45]. Notably, the most commonly recorded connection between MHC and SSc was the HLA-B8, a DR3-containing haplotype. Interestingly, a high frequency of HLA-DR3 detected in PSC patients was also noted. Similarly, HLA-DRB1, HLA-DRB3, and HLA-DR4 have been suggested to contribute to the coexistence of PSC and IBD [46–49]. In addition, HLA-DR2, HLA-DR3, and IRF5 have been reported as the common susceptibility genes in both PBC and SS [25, 26], further indicating that a common genetic background contributes to the coexistence of AILDs and other extrahepatic autoimmune disorders.

The coexistence of SS and PBC is a major example of the so-called autoimmune clustering, and the underlying mechanism has been relatively well studied. Both of these autoimmune diseases are characterized by the progressive immune-mediated destruction of epithelial tissues, either in salivary and lacrimal glands or in the intrahepatic bile ducts. Antimitochondrial autoantibodies (AMAs) to the E2 subunit of the pyruvate dehydrogenase complex (PDC-E2) are serological hallmarks of PBC, which were detected in 95% of patients with PBC [50–52]. Surprisingly, the PBC autoantigen, PDC-E2, has been demonstrated to be present on the surface of salivary epithelial cells in the salivary glands of PBC patients with SS. Matsumoto et al. [53] found that SS and AILD had a similar immune and inflammatory response, especially CD3^+^ T cells in related organizations, suggesting that the liver and salivary glands, lacrimal glands, or other secreting glands may have the same antigenicity. It is plausible to predict that on the basis of a similar susceptibility gene background environmental triggers (putatively infectious agents and xenobiotics) cause salivary or biliary epithelial cell apoptosis and immune tolerance breakdown to self-antigens which are not protected by PDC-E2, leading to the cellular immune response with predominant CD4^+^ T cell infiltration and the mucosal immune response mediated by IgA [25, 26, 33, 38]. Therefore, common susceptibility gene backgrounds are involved in the pathophysiological pathways between PBC and SS.

Last but not least, autoimmune polyendocrine syndrome type 1 (APS-1) should be mentioned. It is a rare monogenetic recessive disorder caused by mutations in the autoimmune regulator (Aire) gene. Patients with APS-1 always developed SS, anemia, diabetes, alopecia, vitiligo, gastritis, and AIH, the last one affecting up to 20% of APS-1 patients [54], which provides a possible connection between AILD and some endocrine-associated autoimmune diseases. To summarize, common susceptibility genes, environmental triggers, and antigen cross-reaction cooperatively contribute to a possibly shared pathogenesis which is involved in the coexistence of AILDs and EHAIDs.

## 6. Conclusion

Commonly, more than one autoimmune condition can occur in same AILD patient. Herein, we discuss EHAIDs in patients with AILDs, particularly in relation to the clinical impact and pathophysiology of these diseases. The incidence of EHAIDs in AILDs and onset time of them can be different. These discrepancies might be explained by different geographical and genetic backgrounds between studies. Importantly, autoimmunity clustering frequently increases the difficulty of diagnosis. In particular, biochemical liver abnormalities in patients with CTDs are common, which may be the result of previous treatments with potentially hepatotoxic drugs or CTDs associated nonspecific liver involvement. Liver biopsy is crucial in distinguishing AILDs in the course of EHAIDs from CTDs associated or drug-induced liver injuries. In our review, it is worth mentioning that we first summarize and analyze a few researches regarding clinical features and impact of concomitant EHAIDs on AILDs. When overlapping with other extrahepatic autoimmune diseases, patients with AILDs manifest special clinical and laboratory features which may have an effect on the natural course and prognosis of AILDs. Certain EHAIDs may aggravate the systemic inflammatory response and liver damage, and others may alleviate the liver inflammatory response, consequently achieving a better prognosis. Furthermore, some multicenter researches with a large sample size and different population background are needed to explore and verify these findings. Finally, we try to explore the pathophysiological pathway which contributes to the coexistence of AILDs and EHAIDs. Currently, it is widely recognized that a common susceptibility genetic background involves the autoimmunity clustering. Therefore, in managing patients with AILDs, gastroenterologists should be aware of concomitant EHAIDs to ensure a prompt diagnosis and better outcome. Future researches should pay more attention to the relationship between genomics and immune regulator factors and confirm a common and distinct pathway involved in the pathogeneses of autoimmunity clustering.

## Figures and Tables

**Table 1:** Incidence of concomitant EHAIDs in AILDs.

	EHAIDs	Sjogren's syndrome	Autoimmune thyroid disease	Systemic lupus erythematosus	Rheumatoid arthritis	Systemic sclerosis or scleroderma	Inflammatory bowel disease	Dermatomyositis or polymyositis	Raynaud's phenomenon	Mixed connective tissue disease	Autoimmune thrombocytopenic purpura	Pernicious anemia	References
AIH	29.9–61.8%	1.4–34.5%	10.0–23.0%	0.7–18.8%	1.8–12.9%	1.2–3.5%	2.0–8.0%	3.6%	—	2.0–4.0%	1 case	—	[3–7]
PBC	36.5–67.4%	3.5–47.4%	14.4–23.8%	1.0–5.2%	1.8–17.0%	0.8–12.3%	2.0–7.5%	0.6–3.1%	18.0–24.0%	0.6–0.8%	1.0%	4.0%	[3, 8–11]
PBC-AIH OS	25.0–43.7%	8.5–20.8%	18.3%	2.8%	4.2%	1.4%	—	1 case	—	—	1 case	1.4%	[12–14]
PSC	60.0–80.0%	2 cases	7.6%	2 cases	5.6%	1 case	1.7–70.0%	—	—	—	—	—	[15–18]

EHAID: extrahepatic autoimmune disease; AILD: autoimmune liver disease; AIH: autoimmune hepatitis; PBC: primary biliary cirrhosis; PSC: primary sclerosing cholangitis; OS: overlap syndrome.

**Table 2:** Liver involvement in CTDs.

	Liver involvement	AIH	PBC	PSC	References
Systemic lupus erythematosus	3.0–79.0%	2.7–20.0%	2.7–15.0%	1 case	[1, 19–29]
Sjogren's syndrome	7.0–49.0%	6.0–47.0%	35.0–57.0%	11 cases	[22, 29–31]
Systemic sclerosis or scleroderma	1.1%	11 cases	51.2%	51.2%	[22, 29, 32–34]
Antiphospholipid syndrome	—	5 cases	1 case	1 case	[13, 35–37]
Dermatomyositis or polymyositis	—	7.1%	14.3%	—	[13, 38]

AIH: autoimmune hepatitis; PBC: primary biliary cirrhosis; PSC: primary sclerosing cholangitis.
